# Hearing Aid Use is Associated with Faster Visual Lexical Decision

**DOI:** 10.1177/23312165251375892

**Published:** 2025-09-19

**Authors:** Ruijing Ning, Carine Signoret, Emil Holmer, Henrik Danielsson

**Affiliations:** Department of Behavioral Sciences and Learning, 4566 Linköping University, Linköping, Sweden

**Keywords:** hearing aid use, hearing loss, hearing impaired, language skill, word recognition, phonological processing

## Abstract

This study investigates the impact of hearing aid (HA) use on visual lexical decision (LD) performance in individuals with hearing loss. We hypothesize that HA use benefits phonological processing and leads to faster and more accurate visual LD. We compared the visual LD performance among three groups: 92 short-term HA users (<5 years), 98 long-term HA users, and 55 nonusers, while controlling for hearing level, age, and years of education. Results showed that, compared with non-HA users, HA users showed significantly faster reaction times in visual LD, specifically, long-term HA use was associated with smaller difference in reaction time for pseudowords compared to nonwords. These results suggest that HA use is associated with faster visual word recognition, potentially reflecting enhanced cognitive functions beyond auditory processing. These findings point to possible cognitive advantages linked to HA use.

## Introduction

Some evidence shows that hearing loss (HL) is negatively associated with visual word recognition (Andersson & Lyxell, 1999; Lyxell et al., 1996, 1998). This impairment may be due to a deterioration in phonological processing caused by reduced and degraded auditory input. Hearing aid (HA) use improves the quality of auditory input, but does it also facilitate visual word recognition through enhanced phonological processing? The current study examined this research question in individuals with HL that had different usage duration of HA.

HL is a prevalent sensory impairment affecting millions of individuals worldwide. Beyond its extensively documented impact on auditory perception, recent research increasingly acknowledges its association with cognitive function (e.g., Lin et al., 2013). Among the cognitive abilities that have been linked to HL, visual word recognition, as measured by visual lexical decision (LD), stands out as an essential component of language processing (Pittman et al., 2017). Visual LD tasks evaluate participants’ ability to quickly recognize whether a series of letters (visually presented) constitutes a word or not.

The processing involved in visual LD differs according to the type of stimuli—the letter strings could be words, nonwords—letter strings that are not pronounceable, or pseudowords—pronounceable letter strings but meaningless (Signoret et al., 2011). When the stimulus is a nonword, the illegal orthography is often sufficient to prompt a negative response. Conversely, when the stimulus is a word or pseudoword, the mental lexicon is accessed and searched. The search process terminates upon locating a matching entry for words, whereas for pseudowords, termination occurs upon eliminating similar entries. Prior investigations have demonstrated different performance in visual LD tasks across the stimulus types: nonwords and words show higher accuracy and shorter reaction times, and pseudowords show lower accuracy and longer reaction times (e.g., Barca & Pezzulo, 2012). In general, visual LD involves accessing the mental lexicon stored in long-term memory (LTM). As this process implicates phonological processing, visual LD tasks also reflect the involvement of a phonological component (Stille et al., 2020). In fact, according to interactive (or connectionist) models of word recognition, higher-order linguistic representations—such as phonology and semantics—can influence early orthographic processing (McClelland & Rumelhart, 1981), suggesting that even responses to nonwords may involve some phonological component.

Research has demonstrated that HL was associated with worse performance on visual LD. Andersson and Lyxell (1999) found that adults with acquired severe HL showed a significantly longer reaction time and lower accuracy in visual LD specifically for pronounceable but meaningless letter strings (i.e., pseudowords). Additionally, Lyxell et al. (1996, 1998) observed that adults who were deafened postlingually exhibited lower accuracy levels in visual LD for pseudowords, but similar reaction time compared with age-matched normal hearing controls. The worse visual LD performance could be associated with reduced efficiency and accuracy in the search of the mental lexicon, potentially linked to HL. Long and repeated exposure to degraded auditory input signal may contribute to changes in the integrity of phonological representations—the mental representation of the sounds and combinations of sounds that comprise a word. As a result, retrieving and recognizing words may be more challenging for individuals with HL, which can negatively affect visual LD task performance. This notion was supported by evidence indicating that HL is negatively associated with other tasks that require phonological processing, such as visual rhyme judgment which requires participants to judge whether two visually presented words rhyme with each other (Classon et al., 2013; Lazard & Giraud, 2017; Rudner et al., 2019). Pseudowords showed the strongest association with HL, potentially because phonological representation played a more important role in searching and eliminating items from mental lexicon than in processing words or nonwords.

This aligns with the Development Ease of Language Understanding (D-ELU) model, which provides a framework for understanding how language input is processed and understood in a changing cognitive system (Holmer et al., 2016). Building on the ELU model (Rönnberg et al., 2022), the D-ELU model states that comprehension of language in any modality involves the matching of sensory input against phonological, lexical, and syntactic representations stored in LTM. Successful matching triggers the rapid, automatic retrieval of corresponding lexical entries (words) from the mental lexicon, while a mismatch requires slower, conscious, and effortful processing. Progressive and untreated HL, in which the sensory signals are constantly degraded, means that there is an increased risk of mismatch with LTM representations, and this pushes the system toward change: restructuring of existing or adding novel phonological representations. HL also means that the change to the system at one time might not help at the next time since the sensory input is continuously changing. Phonological representations in LTM will gradually change but might not become fixed as exemplars that can be used for efficient processing (Rönnberg et al., 2019). The weakened phonological representations would thus hinder the matching of visual input in the visual LD task, leading to decreased accuracy and increased latency. This notion is further supported by findings that HA and cochlear implant use may be associated with better performance in phonological processing tasks (Boudia et al., 1999; Chin & Pisoni, 2000; Ning et al., 2025).

HAs improve auditory perception by amplifying sound and are commonly used in the management of HL. Recent research suggests that, beyond enhancing audibility, HAs are associated with slower rates of cognitive decline (Bucholc et al., 2022; Lin et al., 2023) and improved cognitive functions (Ng & Rönnberg, 2020; Ning et al., 2025). In the present study, we reasoned that because HA use improves the quality of auditory input, it might potentially help to recover the integrity of phonological representations in the mental lexicon, leading to improvement in visual LD performance. This also aligns with the D-ELU model, which posits that consistent HA use leads to the stabilization of phonological representations (Rönnberg et al., 2019, 2022). In addition, longer HA experience has been found to be more beneficial in speech recognition and other aspects of language processing (Kılıç & Kara, 2023; Ng & Rönnberg, 2020; Ning et al., 2025; Turner et al., 1996; Vogelzang et al., 2021), leading to our prediction that long-term HA use might be associated with more improvements in visual LD.

The current study aimed to examine the hypothesis proposing that HA use leads to improved visual LD performance, by testing whether visual LD precision and processing speed was associated with degree of HA use in individuals with HL. We took advantage of a dataset (n200) that included non-HA users with HL and HA users with different duration of use (Rönnberg et al., 2016). We predicted that HA users would show a better visual LD performance (higher accuracy and shorter latency) than non-HA users when controlling for other factors that could affect visual LD such as hearing level, working memory capacity (WMC), fluid intelligence, age, and years of education (Kosmidis et al., 2006), and that long-term HA use will be associated with more improvements in visual LD performance. Both WMC and fluid intelligence were included as covariates to account for their contributions to cognitive processing, with WMC reflecting the ability to temporarily store and manipulate information (Baddeley et al., 1975), and fluid intelligence reflecting the ability to reason and to “construct meaning out of confusion” (Raven, 2008). Moreover, we also predicted that the effect of HA use would be more obvious for pseudowords than words, and least obvious for nonwords, since the processing of words and pseudowords rely more on phonological representations than nonwords, and that pseudowords showed more deterioration caused by HL than words.

## Method

### Participants

The data presented in this study is part of the n200 database (Rönnberg et al., 2016). In this database, a total of 286 individuals with HL are included, of which 215 are hearing-aid users with bilateral, symmetrical sensorineural HL in the range mild-to-severe (the HA group), 71 have bilateral, symmetrical sensorineural HL in the range mild-to-severe but do not use HAs (the NoHA group). To evaluate the association between HA use and LD, we divided the HA group into two subgroups based on the duration of their HA use, with the median of HA use as the cut point. This resulted in three groups (NoHA, ShortHA, and LongHA). The ShortHA and LongHA groups were recruited from the patient population at the Linköping University Hospital. The NoHA group was recruited via mail based on information from SCB (Statistics Sweden) as normal-hearing controls but turned out to have HL. All participants gave informed consent and were native Swedish speakers with normal or corrected-to-normal vision, and no reports of neurological diagnoses. The study was approved by the Linköping regional ethical review board (Dnr: 2016/418-32).

### Procedure and Task

As part of the n200 project, participants underwent a battery of tests regarding hearing, cognitive, and executive functions distributed in three testing sessions. The tests relevant to this paper, the LD task and the Raven's test, were carried out on the first session (for details, see Rönnberg et al., 2016).

#### The LD Task

Materials consist of three-letter sequences that were presented on the computer screen. The participants were asked to judge whether a letter string constituted a real word or not (e.g., Rönnberg, 1990). Forty items were used of which 20 were common Swedish words (word frequency in Zipf value: mean = 3.85, SD = 1.28, according to Witte & Köbler, 2019), 10 were pseudowords, and 10 were nonwords. The participants’ task was to respond “yes” (for a real word) or “no” (for a nonword/pseudoword) by pressing physical buttons during a 5,000 ms interval before the presentation of the next string of letters. A green physical button indicated “yes,” and a red button indicated “no,” with the button positions remaining constant throughout the experiment. The participants were instructed to respond as quickly and accurately as possible. The accuracy and reaction time were recorded for data analysis. Reaction time was measured as the interval between the onset of the letter string presentation and the participant's button press. Only reaction times for the correct trials were entered into data analysis, as these are more indicative of task-related processing.

#### The Raven's Test

Raven's standard progressive matrices (e.g., Raven, 2008) was used to measure fluid intelligence. Three (A, D, and E) out of five sets of the Raven test were administered. Set A was used for practice, and the experimenter could give feedback to the participant. Sets D and E, 12 items each, were administered on paper without feedback or time limit. In each item, there is a missing piece in a pattern. The participant was asked to choose one out of the six alternatives which completes the overall pattern. The participant responded by marking with a pencil on a scoring sheet. The test was scored by the sum of points (max 24).

#### The Reading Span Test

A Swedish version of Daneman and Carpenter (1980) Reading Span test was used to assess WMC (Rönnberg et al., 1989). The test involved sets of two to five sentences, displayed one word at a time on a computer screen, with each word shown for 800 ms. After reading each sentence, participants indicated whether it was logically coherent or nonsensical. Following each set, they were instructed to recall either the initial or final word of every sentence in the correct sequence. A total of 28 sentences were presented. The test was scored by the total number of items correctly recalled irrespective of recall order.

### Data Analysis

To test the predictions of this study, we conducted analyses focusing on the difference in visual LD performance in individuals with hearing impairment related to HA use. We conducted two generalized linear mixed models (GLMMs) using the accuracy of each trial as the dependent variable in the first model, and the reaction time of each correct trial (in s) as the dependent variable in the second model. Accuracy was fitted using binomial distribution, while reaction time using inverse Gaussian distribution (identity link). Inverse Gaussian was chosen because the data had a right skew (skewness = 3.359), and it had a better fit than using a gamma distribution and linear model (Lo & Andrews, 2015). Group and stimulus type (word, pseudoword, or nonword), and their interaction were entered in the model as fixed effects. Both *group* and *stimulus type* were specified as factor variables. Following the treatment coding, *NoHA* was treated as the reference level for *group* (coded as 0), and *nonword* was treated as the reference level (0) for stimulus type. This coding allowed the model to estimate the effects of HA use (*ShortHA* or *LongHA*) relative to *NoHA*, and lexical status (*word* or *pseudoword*) relative to *nonword*, along with their interaction. Age, better-ear pure tone average (PTA), years of education, reading span test score, and Raven's test score were scaled and centered, and entered into the model as covariates. Individual participants and visual LD items were entered as random effects (intercepts only). Models with by-item random slopes for group or stimulus type were tested in likelihood ratio tests against the random intercept only model, but did not significantly improve the model fit. Thus, we chose to retain the simpler model with random intercepts only. Visual inspection of residuals and random effects suggested no major violations of model assumptions, indicating an adequate model fit. For the GLMMs, posthoc analysis with the False Discovery Rate adjustment was conducted to test between group contrasts at specific levels of stimulus type. The data analysis was carried out in R with packages including lme4 (Bates et al., 2015) and emmeans (Lenth et al., 2023).

## Results

Participants who did not complete the LD test (*n* = 14) were excluded from further analysis. Participants who had missing value in better-ear PTA, Reading Span test score, Raven's test score, or years of education (*n* = 13) were also excluded. Participants in the HA group who did not report the duration of their HA use (*n* = 15) were excluded. Single trials that had a reaction time shorter than 100 ms were removed, because these quick reaction times in the population being tested usually result from mistakes in button pressing. As a result, two trials by two participants were removed.

After the exclusion, 244 participants and a total of 9,730 trials were included in the analysis (the HA group: *n* = 190, 87 females; the NoHA group: *n* = 54, 24 females). For the HA group, a median split based on HA experience, resulted in a “LongHA” group with participants who used HA longer than or equal to 5 years (*n* = 98, 44 females), and a “ShortHA” group consisting of participants who used HA shorter than 5 years (*n* = 92, 43 females). See Table 1 for the descriptive statistics of the variables involved in the current analysis. As expected, the NoHA group had lower average better-ear air-conduction PTA at 0.5, 1, 2, and 4 kHz than the ShortHA and LongHA groups (*t*(241) ≤ −2.470, *p* ≤ .038). The ShortHA group's average better-ear PTA was significantly lower than that of the LongHA group (*t*(241) = −6.620,  < .001). See Table 1 for pair-wise comparisons on other variables among groups.

**Table 1.** Descriptive Statistics and Comparisons Among the Three Groups in the Variables Involved in the Current Analysis.

					Comparison (*t*-ratio)		
	Total	NoHA	ShortHA	LongHA	NoHA–ShortHA	ShortHA–LongHA	NoHA–LongHA
	(*N* = 244)	(*N* = 54)	(*N* = 92)	(*N* = 98)			
Hearing aid use (years)							
Mean (SD)	5.37 (6.62)	0.00 (0.00)	2.36 (1.00)	11.15 (7.10)	−3.03**	−13.32***	−14.47***
Lexical decision (% correct)							
Mean (SD)	0.97 (0.04)	0.97 (0.04)	0.97 (0.03)	0.97 (0.05)	0.05	0.64	0.6
Nonword	0.99 (0.11)	0.99 (0.03)	0.99 (0.03)	0.99 (0.04)	−0.23	0.72	0.38
Word	0.98 (0.15)	0.97 (0.03)	0.98 (0.04)	0.98 (0.04)	0.06	−0.73	−0.56
Pseudoword	0.94 (0.25)	0.94 (0.09)	0.94 (0.12)	0.92 (0.17)	0.02	0.99	0.87
Lexical decision (response time, ms)							
Mean (SD)	991 (202)	1,062 (231)	962 (184)	980 (193)	2.93*	−0.61	2.44*
Nonword	851 (269)	820 (142)	915 (207)	846 (165)	3.31**	−1.09	2.42*
Word	932 (393)	901 (161)	1,004 (238)	923 (179)	3.19**	−0.82	2.52*
Pseudoword	1,249 (577)	1,231 (386)	1,330 (338)	1,228 (363)	1.56	0.07	1.64
Age (years)							
Mean (SD)	63.06 (8.43)	68.85 (7.39)	59.61 (7.87)	63.10 (7.74)	6.99***	−3.12**	4.4***
Better-ear PTA (dB HL)							
Mean (SD)	35.46 (10.80)	28.73 (6.35)	32.70 (10.06)	41.75 (10.15)	−2.47*	−6.62***	−8.16***
Education (years)							
Mean (SD)	13.09 (3.37)	12.15 (2.88)	13.55 (3.23)	13.17 (3.67)	−2.45*	0.79	−1.8
Reading Span (no. of correct recall, max 28)							
Mean (SD)	15.48 (3.90)	14.06 (3.90)	16.09 (3.51)	15.69 (4.10)	−3.08**	0.7	−2.52*
Raven test (correct responses, max 24)							
Mean (SD)	14.80 (4.90)	13.15 (5.38)	15.59 (4.69)	14.97 (4.63)	−2.95**	0.88	−2.23

*Note*. dB HL = decibels Hearing Level.

***p (Tukey) < .001; **p (Tukey) < .01; *p (Tukey) < .05.

### LD Accuracy

The NoHA, ShortHA, and LongHA groups performed similarly on visual LD accuracy (see Table 2; accuracy group: | $\hat{\beta }$ | ≤ 0.58, |*z*| ≤ 1.04, *p* ≥ .298), regardless of the stimulus type (see Table 2; accuracy group × Type: | $\hat{\beta }$ | ≤ 0.35, |*z*| ≤ 0.62, *p* ≥ .537). In terms of stimulus type, nonwords showed similar accuracy with words (see Table 2; Accuracy stimulus type [Word]: $\hat{\beta }$ = −0.52, *z* = −0.82, *p* = .412), but significantly better accuracy than pseudowords (see Table 2; Accuracy stimulus type [Pseudoword]: $\hat{\beta }$ = −1.92, *z* = −2.88, *p* = .004). Words also showed better accuracy than pseudowords (posthoc comparison: mean difference = −0.028, *z* = −2.37, *p* = .027).

**Table 2.** Summary of the Two GLMMs for LD Accuracy and Reaction Time.

	Accuracy				Reaction time			
Predictors	$\hat{\beta }$	95% CI	*z*	*p*	$\hat{\beta }$	95% CI	*t*	*p*
Intercept	5.64	[4.51, 6.78]	9.73	**< .001**	1,092.52	[1,054.28, 1,130.77]	55.99	**< .001**
Group[ShortHA]	−0.23	[−1.37, 0.90]	−0.41	.684	−63.1	[−92.85, −33.34]	−4.16	**< .001**
Group[LongHA]	−0.58	[−1.66, 0.51]	−1.04	.298	−63.08	[−92.10, −34.06]	−4.26	**< .001**
Stimulus type[Word]	−0.52	[−1.76, 0.72]	−0.82	.412	90.4	[49.70, 131.10]	4.35	**< .001**
Stimulus type[Pseudoword]	−1.92	[−3.23, −0.62]	−2.88	.**004**	258.92	[233.56, 284.28]	20.01	**< .001**
Age	0.08	[−0.13, 0.30]	0.76	.446	−12.73	[−33.26, 7.80]	−1.22	.224
PTA	0.24	[0.03, 0.44]	2.24	.**025**	31.99	[12.44, 51.54]	3.21	.**001**
Years of education	0.35	[0.14, 0.57]	3.21	.**001**	−9.42	[−29.64, 10.80]	−0.91	.361
Reading Span test	0.26	[0.06, 0.45]	2.57	.**01**	−53.86	[−75.12, −32.60]	−4.97	**< .001**
Raven test	0.04	[−0.17, 0.26]	0.4	.689	−26.37	[−48.27, −4.47]	−2.36	.**018**
Group[ShortHA] × Type[Word]	−0.12	[−1.29, 1.06]	−0.19	.847	4.54	[−14.81, 23.89]	0.46	.646
Group[LongHA] × Type[Word]	0.35	[−0.77, 1.48]	0.62	.537	−3.9	[−23.11, 15.32]	−0.4	.691
Group[ShortHA] × Type[Pseudoword]	−0.13	[−1.29, 1.03]	−0.21	.831	−12.13	[−35.13, 10.86]	−1.03	.301
Group[LongHA] × Type[Pseudoword]	−0.2	[−1.29, 0.89]	−0.36	.721	−35.98	[−58.25, −13.71]	−3.17	.**002**

*Note*. GLMM = generalized linear mixed model; LD = lexical decision; CI = confidence interval; PTA = pure tone average. Significant *p*-values are shown in bold.

### LD Reaction Time

**Figure 1.**
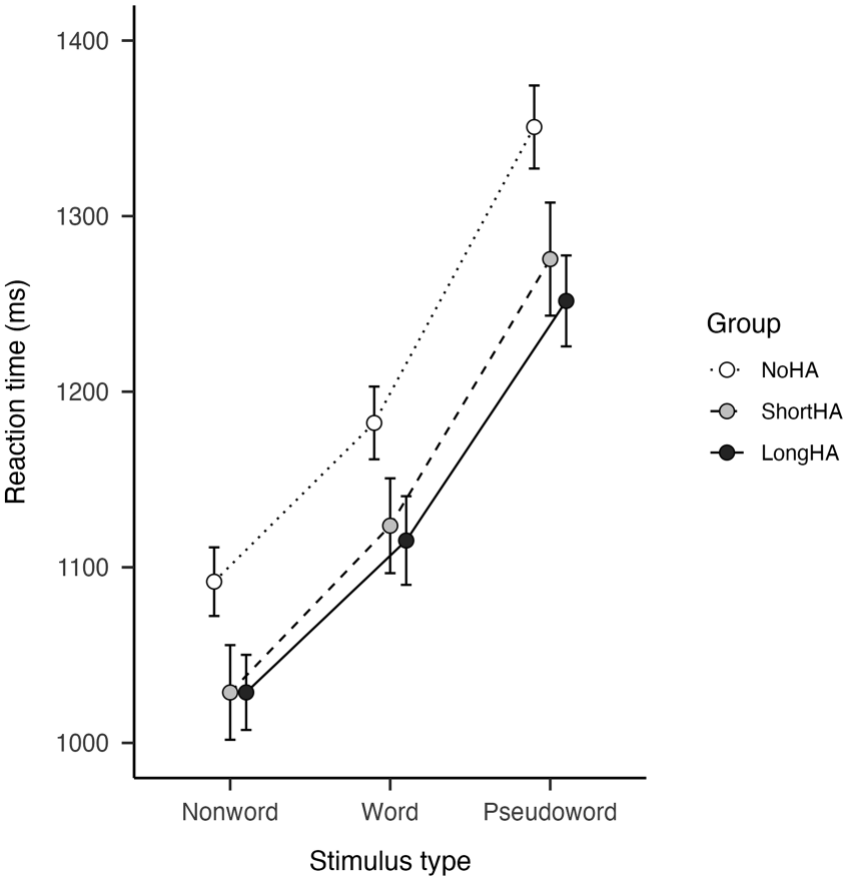
Estimated reaction times for the visual LD task, plotted by group and stimulus type. Values represent model estimates from the GLMM, adjusted for covariates. Error bars indicate ±1 standard error (SE). LD = lexical decision; GLMM = generalized linear mixed model.

The NoHA, ShortHA, and LongHA groups showed different visual LD reaction times. Importantly, the NoHA group had a significantly longer reaction time than ShortHA and LongHA groups across all stimulus types when controlling for covariates (see Figure 1 and Table 2; Reaction time group: | $\hat{\beta }$ | ≥ 63.08, |*t*| ≥ 4.16, *p* ≤ .001). However, there was no significant difference between the ShortHA and LongHA groups (posthoc comparison: mean difference = 10.70, *z* = 0.53, *p* = .597, Cohen's *d* = 0.10).

In terms of stimulus types, pseudowords showed significantly longer reaction time than words (posthoc comparison: mean difference = 152.30, *z* = 6.82, *p* < .001, Cohen's *d* = 1.36), which in turn showed longer reaction time than nonwords (see Table 2; Reaction time stimulus type [Word]: $\hat{\beta }$ = 90.40, *t* = 4.35, *p* < .001).

The difference in reaction time between nonwords and pseudowords was on average 35.98 ms smaller in the LongHA than the NoHA group (see Figure 1 and Table 2; Reaction time group [LongHA] × Type [Pseudoword]: *t* = −3.17, *p* = .002). This interaction between group and stimulus type suggests that longer HA use might be particularly beneficial for the processing of pseudowords. This difference, however, did not result in a significant difference between the reaction time required for pseudowords between the ShortHA and LongHA groups (posthoc comparison: mean difference = 23.80, *z* = 1.04, *p* = .383, Cohen's *d* = 0.21).

## Discussion

The present study investigated the association between HA use and LD performance in individuals with HL. We focused on evaluating the visual LD performance in long-term (5 years or longer: LongHA) and short-term HA users (ShortHA) compared to hearing-impaired non-HA users (NoHA). We found that controlling for age, hearing level, WMC, fluid intelligence, and years of education, the HA users showed shorter visual LD reaction time but not higher accuracy than the nonusers; long-term HA use was associated with more decrease in reaction time for pseudowords compared to words.

Our analysis showed no significant difference in visual LD accuracy across the NoHA, ShortHA, and LongHA groups. This outcome suggests that the use of HAs, regardless of duration, was not markedly associated with the ability to accurately discern between words, pseudowords, and nonwords in the current experimental setting. A similar finding was reported for cochlear implants in deafened adults, that the visual LD accuracy did not improve 1 year after cochlear implant (Lyxell et al., 1998). It may be that visual LD accuracy is not sensitive to hearing interventions, possibly due to a ceiling effect, as the very high overall accuracy across groups could have limited the task's ability to detect group differences. Nevertheless, this finding does not lend support to our hypothesis that HA use improved the precision of visual LD through stabilizing phonological representations.

The reaction time results indicated that HA use was associated with the speed of visual LDs, as indicated by the main effect of group in the model. Both ShortHA and LongHA groups exhibited faster reaction times compared to the NoHA group. This finding is consistent with our hypothesis based on the D-ELU model that HA use leads to the stabilization of phonological representations (Rönnberg et al., 2022), facilitating the matching of visual input in visual LD on to entries in the mental lexicon. The finding also suggested that the visual LD accuracy in the NoHA group was maintained with a cost of slower processing.

Both the accuracy and reaction time results indicated an effect of stimulus type, as evidenced by a significant main effect of stimulus type in the models. Pseudowords were less accurately identified than words and nonwords. Pseudowords also had longer reaction times, aligning with previous research indicating the complexity of processing pseudowords due to their phonological similarity to actual words (Barca & Pezzulo, 2012). Words showed similar accuracy but elicited longer reaction times than nonwords. This difference in reaction time could be due to the choice of stimuli for words in the current study. It was reported that the reaction time for words varied based on the word frequency: the reaction time for high-frequency words could be as short as nonwords, but the reaction time for low-frequency words was considerably longer (e.g., Barca & Pezzulo, 2012). The words used in the current study had a mean Zipf frequency of 3.85, which corresponds to moderately low frequency, potentially contributing to the longer reaction times observed.

Notably, HA use was associated with faster reaction times in all three stimulus types, while long-term HA use, in particular, showed a stronger association with the reaction times of pseudowords compared to nonwords, as indicated by the interaction term in the model. This finding aligns with our prediction that the effect of HA use would be mainly observed for pseudowords. Words did not show this difference, potentially because words are not as sensitive to changes in phonological representation (Andersson & Lyxell, 1999; Lyxell et al., 1996, 1998). This could be explained using the ELU framework (Rönnberg et al., 2013, 2019) stipulating that, in the matching process, words are processed faster than pseudowords due to the semantic support in LTM. Beyond the ELU framework, these results can also be integrated with general models of visual word recognition. According to interactive accounts, phonological and lexical information can influence orthographic processing at early stages of word recognition (Carreiras et al., 2014). This is in line with our finding that reaction times for nonwords were also faster in HA users when controlling for covariates. Moreover, our finding that long-term HA use had a stronger effect on pseudowords than nonwords may thus reflect plastic changes in semantic integration following HL.

Despite the significant interaction between group and stimulus type, the ShortHA and LongHA groups did not show significant differences in reaction times in any stimulus type. Apart from this being an issue of statistical power, this outcome could be interpreted to suggest (1) the association between HA use and visual LD took shorter than 5 years to emerge, therefore the current cut point of 5 years did not catch the transition; or (2) the association between of long-term HA use and pseudowords and words could have been washed down by longer HL history. Even when the hearing level was controlled, longer HL may have negatively impacted their phonological representations (Classon et al., 2013). The current study did not provide further evidence regarding the two possibilities. Thus, a closer look at the association between HA use and visual LD while considering individuals’ hearing impairment history would be needed to reveal the time course of the potential benefit of HA use.

The results presented in Table 2 indicate potential main effects of WMC (Reading Span) and fluid intelligence (Raven's test score) on visual LD performance. However, these findings should be interpreted with caution, as the estimates were limited to the baseline condition (the NoHA group in the nonword condition). This limitation stems from our decision to include WMC and fluid intelligence as covariates in order to control for their influence and isolate the effects of primary interest. As a result, interactions involving these cognitive factors were not modeled, which limits our ability to draw conclusions about their roles in task performance across different groups or conditions. Since it is plausible that WMC and fluid intelligence interact with HA use in tasks involving phonological processing (Ng & Rönnberg, 2020; Ning et al., 2025), future research should explicitly examine such interactions to better understand how cognitive and auditory systems jointly adapt to changing listening environments. A limitation of this study lies in its cross-sectional and observational design, which restricts the ability to infer causality. While we proposed that the outcome indicates that HA use benefits LD performance, it is also plausible that those with faster lexical access are more inclined to use HAs. Future research adopting longitudinal or randomized controlled design could provide stronger causal evidence for the advantages of HAs by revealing whether (prolonged) HA use leads to (accumulative) cognitive benefits.

Another limitation of the study is the group differences in chronological age, WMC, and fluid intelligence, with the NoHA group being older and scoring lower on the cognitive tests than the HA groups. Although these factors were statistically controlled, residual confounding cannot be ruled out, particularly given the potential for complex interactions not fully captured by our models. Therefore, the differences in reaction times may reflect the general processing speed rather than phonological processing. In addition, the duration of HL prior to HA use was not available, which may have influenced performance and limited interpretation of the differences—or lack thereof—between ShortHA and LongHA users. Future research with more closely matched groups, larger samples, and detailed hearing history could help clarify these relationships and further disentangle the effects of HA use, age, and cognition on phonological processing.

It should be acknowledged that the present study did not control for specific lexical properties of the stimuli, including orthographic and phonological neighborhood density and phonotactic probability. These factors were not matched across stimulus types (words, pseudowords, and nonwords), as the LD task was based on an established design that has been used in several previously published studies (e.g., Classon et al., 2013; Rönnberg, 1990). Importantly, as group comparisons were modeled with item as a random effect, variability at the item level should have been partly accounted for. Moreover, achieving such matching is not always realistic especially between nonwords and other stimuli: nonwords in this task were constructed to violate Swedish phonotactic rules and are therefore likely to have very low phonotactic probability and few, if any, lexical neighbors. Nevertheless, it is worth noting that matching pseudowords with words on neighborhood density and phonotactic probability would be meaningful as it has been demonstrated that higher neighborhood density can facilitate processing of real words but inhibit recognition of pseudowords (Grainger & Jacobs, 1996). Therefore, future work using recent lexical databases (e.g., Witte & Köbler, 2019) could help disentangle the contributions of individual lexical characteristics to task performance.

A methodological limitation of the study lies in the use of a median split to classify participants as short- or long-term HA users. While this approach was chosen for descriptive clarity and to balance group sizes, it introduces an arbitrary cutoff that may obscure meaningful individual differences. A visual inspection of the plot of the continuous variables showed no major discrepancies among participants either below or above the median, suggesting that a median split was appropriate. To complement this approach, we conducted supplementary analyses treating HA use as a continuous variable (log-transformed). This did not reveal a significant negative linear effect on response time, but became significant with the addition of a quadratic term, suggesting a possible nonlinear (U-shaped) trend (see Supplemental Results). However, model comparison did not show a clear advantage for the quadratic model. Given the data's strong skew toward shorter HA durations, the power to detect continuous effects may have been limited. Future research would benefit from modeling HA use continuously within a more evenly distributed sample, and ideally using longitudinal data to clarify nonlinear adaptation trajectories over time.

In summary, this study demonstrated that HA use was associated with enhanced speed in visual LD in individuals with HL, supporting the hypothesis that HA use helps word recognition through stabilizing phonological representations in LTM, and suggests a possible impact of HA use on cognitive functions related to language processing.

## Supplemental Material

sj-docx-1-tia-10.1177_23312165251375892 - Supplemental material for Hearing Aid Use is Associated with Faster Visual Lexical Decision Supplemental material, sj-docx-1-tia-10.1177_23312165251375892 for Hearing Aid Use is Associated with Faster Visual Lexical Decision by Ruijing Ning, Carine Signoret, Emil Holmer and Henrik Danielsson in Trends in Hearing
