# Supplementary material for: Hearing Aid Use is Associated with Faster Visual Lexical Decision
Source: Trends Hear. 2025 Sep 19;29:23312165251375892. doi: 10.1177/23312165251375892 (PMC12449647; doi:10.1177/23312165251375892)
Supplement: sj-docx-1-tia-10.1177_23312165251375892 - Supplemental material for Hearing Aid Use is Associated with Faster Visual Lexical Decision [file sj-docx-1-tia-10.1177_23312165251375892.docx]

# **Supplementary Analyses and Results:**

# **Hearing aid use as a continous variable**

To explore the effect of hearing aid (HA) use as a continuous variable, we reanalyzed the data using the log-transformed number of years of HA use as a predictor. A log(+1) transformation was applied to accommodate zero values and address the skewed distribution of HA use duration (skewness = 2.46). This transformation also reflects a theoretically plausible model of diminishing returns in HA adaptation, where benefits may accrue more rapidly in early years and level off over time (Dosher & Lu, 1998; Goldstone, 1998).

For both accuracy and reaction time (RT), we fit generalized linear mixed models (GLMMs). The RT model used an inverse Gaussian distribution with identity link, and the accuracy model used a binomial distribution, consistent with the models reported in the main analysis. Fixed effects included log-transformed years of HA use, stimulus type, their interaction, and covariates (age, PTA, years of education, reading span, and Raven’s test score). Due to convergence issues in more complex models, the random effects structure was simplified to include random intercepts for participants only.

Results are presented in Supplementary Table S1. Log-transformed years of HA use did not significantly predict either accuracy or RT, nor did it interact significantly with stimulus type. Modeling years of HA use as a continuous predictor did not yield significant effects (β = -19.86, *p* ≈ .10), which was not consistent with our main analyses revealing a significant difference in reaction time between HA users and non-users. This might be because adaptation to amplification may follow a non‐linear trajectory—rapid gains early on that plateau over time—which a simple log‐linear predictor cannot fully capture. To test this possibility, we included a quadratic term in another model for the reaction time. The model included fixed effects for stimulus type, their interactions with both the linear and quadratic HA use terms, and relevant covariates (age, PTA, education, reading span, Raven’s test score). Random intercepts were included for participants.

Results (See Supplementary Table S2) revealed a significant quadratic effect of HA use (β = 18.53, *p* = .024), suggesting a non-linear relationship between log-transformed HA experience and reaction time. This suggests that reaction times decreased with increasing HA use up to a point, after which they began to rise again. This U-shaped pattern indicates that intermediate levels of HA experience (5-6 years) were associated with the fastest responses, while both shorter and longer durations of use were linked to slower responses.

This non-linear effect was not strong enough to significantly improve model fit over the simpler model (likelihood ratio test, *p* = .14), but it does offer a potential explanation for the weak or non-significant linear trends observed earlier. Future research with more balanced sampling across HA use durations may help clarify this pattern.

Table S1. Summary of the two GLMMs for LD accuracy and reaction time with years of HA use modeled as a continuous variable.

|  | **Accuracy** | | | | **Reaction Time** | | | |
| --- | --- | --- | --- | --- | --- | --- | --- | --- |
| ***Predictors*** | $\hat{\beta}$ | *95% CI* | *z* | *p* | $\hat{\beta}$ | *95% CI* | *t* | *p* |
| Intercept | 4.85 | [4.44, 5.26] | 23.18 | **< .001** | 902.85 | [885.34, 920.36] | 101.05 | **< .001** |
| HA Years (log) | -0.12 | [-0.55, 0.31] | -0.55 | 0.58 | -19.86 | [-43.81, 4.10] | -1.62 | 0.104 |
| Stimulus type[Word] | -0.76 | [-1.18, -0.34] | -3.54 | **< .001** | 67.57 | [57.82, 77.32] | 13.58 | **< .001** |
| Stimulus type[Pseudoword] | -1.84 | [-2.25, -1.43] | -8.76 | **< .001** | 339.28 | [324.23, 354.33] | 44.19 | **< .001** |
| Age | 0.11 | [-0.08, 0.30] | 1.12 | 0.264 | -8.68 | [-32.07, 14.72] | -0.73 | 0.467 |
| PTA | 0.2 | [-0.01, 0.40] | 1.86 | 0.063 | 37.75 | [17.04, 58.45] | 3.57 | **< .001** |
| Years of Education | 0.33 | [0.12, 0.54] | 3.15 | **0.002** | -11.09 | [-33.01, 10.83] | -0.99 | 0.321 |
| Reading Span Test | 0.25 | [0.06, 0.43] | 2.6 | **0.009** | -58.31 | [-75.84, -40.79] | -6.52 | **< .001** |
| Raven Test | 0.03 | [-0.17, 0.24] | 0.32 | 0.748 | -29.74 | [-53.17, -6.31] | -2.49 | **0.013** |
| HA Years(log)*Type[Word] | 0.11 | [-0.32, 0.55] | 0.51 | 0.612 | -8.43 | [-18.21, 1.35] | -1.69 | 0.091 |
| HA Years(log)*Type[Pseudoword] | -0.09 | [-0.52, 0.34] | -0.42 | 0.677 | -10.24 | [-23.87, 3.39] | -1.47 | 0.141 |

Table S2. Summary of the GLMM for LD reaction time with years of HA use modeled as a continuous variable (with quadratic term).

|  | **Reaction Time** | | | |
| --- | --- | --- | --- | --- |
| ***Predictors*** | $\hat{\beta}$ | *95% CI* | *t* | *p* |
| Intercept | 883.739 | 9.923 | 89.062 | **< .001** |
| HA Years (log) | –17.321 | 10.006 | –1.731 | 0.083 |
| [HA Years (log)]^2 | 18.525 | 8.19 | 2.262 | **.024** |
| Stimulus type[Word] | 69.709 | 6.13 | 11.372 | **< .001** |
| Stimulus type[Pseudoword] | 327.29 | 8.34 | 39.245 | **< .001** |
| Age | –14.714 | 7.944 | –1.852 | 0.064 |
| PTA | 32.178 | 10.508 | 3.062 | **.002** |
| Years of Education | –13.035 | 10.63 | –1.226 | 0.22 |
| Reading Span Test | –59.117 | 8.899 | –6.643 | **< .001** |
| Raven Test | –30.520 | 9.955 | –3.066 | **.002** |
| HA Years(log)*Type[Word] | –8.346 | 4.814 | –1.734 | 0.083 |
| HA Years(log)*Type[Pseudoword] | –11.575 | 6.216 | –1.862 | 0.063 |
| [HA Years (log)]^2*Type[Word] | –2.418 | 4.65 | –0.520 | 0.603 |
| [HA Years (log)]^2*Type[Pseudoword] | 12.683 | 6.703 | 1.892 | 0.058 |

**References**

Dosher, B. A., & Lu, Z.-L. (1998). Perceptual learning reflects external noise filtering and internal noise reduction through channel reweighting. *Proceedings of the National Academy of Sciences*, *95*(23), 13988–13993. https://doi.org/10.1073/pnas.95.23.13988

Goldstone, R. L. (1998). Perceptual learning. *Annual Review of Psychology*, *49*, 585–612. https://doi.org/10.1146/annurev.psych.49.1.585
